# EMB6/481: Evidence-Based Patient Education on the Web: Methods and studies for determining consumers' needs

**DOI:** 10.2196/jmir.1.suppl1.e24

**Published:** 1999-09-19

**Authors:** G Eysenbach, D Fartasch, TL Diepgen

**Affiliations:** ^1^Institute for Clinical Social Medicine, HeidelbergGermany

**Keywords:** Patient Education, Consumers, Patient-Physician Relationship, Email, Evidence-Based Medicine

## Abstract

**Introduction:**

On the Internet, thousands of medical Web sites aim to educate consumers in the field of health. The quality of the educational material for patients varies greatly and is rarely "evidence-based" - not only in terms of the scientific accuracy but also in terms of sometimes questionable appropriateness for the target group. Material boring the reader with facts already known to him/her and failing to address common questions or misunderstandings will most likely fail to reach its aims. Conventional studies to determine the information needs of patients and their relatives are expensive and difficult to conduct. At our dermatology portal site we conducted two separate studies using two different approaches to determine the "needs" of the consumers by using both most commonly used "feedback channels" the Internet has to offer: Emails and Web forms.

**Methods:**

In the first study, we systematically reviewed 209 unsolicited emails we received from consumers in response to our dermatology Web site, analyzing their contents in respect to previous contacts with live physicians, disease duration, level of frustration expressed in the e-mails, and type of information sought. In another study, we developed a web-based questionnaire as a "pre-test" to determine the knowledge level of patients visiting a patient education site on atopic eczema. The "pre-test" is a form-based "quiz", users are asked to voluntarily complete this questionnaire before entering the information system. The quiz contains 24 statements about atopic eczema, which the user should mark as "true" or "untrue". Once completed, answers are stored in a database and the system generates a dynamic, customized page for the user with explanations and hyperlinks to the respective Web pages which contain further information about the respective items.

**Results:**

Analysis of the 209 emails revealed that many dermatologic patients surfing the Internet and requesting tele-advice have a chronic disease (81%) and seek a second opinion. Seventeen percent express frustration about previous encounters with live physicians. 40% of all e-mails could have been answered by a librarian. In at least 5 instances patients attempt self-diagnosis. Analysis of 1010 responses to the quiz, entered within 5 months (27.2.-27.7.98), revealed some common misunderstandings among patients with atopic eczema, for example that it is a psychosomatic disease or "disease of the nerves", that a special diet is principally required and that AE is an allergy (which is an especially common misunderstanding among parents!).

**Discussion:**

The studies demonstrate how easy, fast and cost-effectively the information needs of patients and consumers can be determined using interactive venues on the Internet and give some insights into the motivation of consumers to consult the Internet.

